# Extracellular Vesicles Released by Tumor Endothelial Cells Spread Immunosuppressive and Transforming Signals Through Various Recipient Cells

**DOI:** 10.3389/fcell.2020.00698

**Published:** 2020-09-09

**Authors:** Tatiana Lopatina, Enrica Favaro, Ludmila Danilova, Elana J. Fertig, Alexander V. Favorov, Luciane T. Kagohara, Tiziana Martone, Benedetta Bussolati, Renato Romagnoli, Roberto Albera, Giancarlo Pecorari, Maria Felice Brizzi, Giovanni Camussi, Daria A. Gaykalova

**Affiliations:** ^1^Department of Medical Sciences, University of Turin, Turin, Italy; ^2^Department of Oncology, The Sidney Kimmel Comprehensive Cancer Center, Johns Hopkins University School of Medicine, Baltimore, MD, United States; ^3^Laboratory of System Biology and Computational Genetics, Vavilov Institute of General Genetics, Moscow, Russia; ^4^Department of Neuroscience “Rita Levi Montalcini”, University of Turin, Turin, Italy; ^5^Department of Molecular Biotechnology and Health Sciences, University of Turin, Turin, Italy; ^6^General Surgery 2U, Liver Transplantation Center, AOU Città della Salute e della Scienza di Torino, University of Turin, Turin, Italy; ^7^Division of Otorhinolaryngology, Department of Surgical Sciences, University of Turin School of Medicine, Turin, Italy; ^8^Department of Otolaryngology – Head and Neck Surgery, Johns Hopkins University School of Medicine, Baltimore, MD, United States

**Keywords:** extracellular vesicles, tumor endothelial cells, tumor immune editing, T regulatory (T reg) cells, head and neck cancer

## Abstract

Head and neck squamous cell carcinoma (HNSCC) has a high recurrence and metastatic rate with an unknown mechanism of cancer spread. Tumor inflammation is the most critical processes of cancer onset, growth, and metastasis. We hypothesize that the release of extracellular vesicles (EVs) by tumor endothelial cells (TECs) induce reprogramming of immune cells as well as stromal cells to create an immunosuppressive microenvironment that favor tumor spread. We call this mechanism as non-metastatic contagious carcinogenesis. Extracellular vesicles were collected from primary HNSCC-derived endothelial cells (TEC-EV) and were used for stimulation of peripheral blood mononuclear cells (PBMCs) and primary adipose mesenchymal stem cells (ASCs). Regulation of ASC gene expression was investigated by RNA sequencing and protein array. PBMC, stimulated with TEC-EV, were analyzed by enzyme-linked immunosorbent assay and fluorescence-activated cell sorting. We validated *in vitro* the effects of TEC-EV on ASCs or PBMC by measuring invasion, adhesion, and proliferation. We found and confirmed that TEC-EV were able to change ASC inflammatory gene expression signature within 24–48 h. TEC-EV were also able to enhance the secretion of TGF-β1 and IL-10 by PBMC and to increase T regulatory cell (Treg) expansion. TEC-EV carry specific proteins and RNAs that are responsible for Treg differentiation and immune suppression. ASCs and PBMC, treated with TEC-EV, enhanced proliferation, adhesion of tumor cells, and their invasion. These data indicate that TEC-EV exhibit a mechanism of non-metastatic contagious carcinogenesis that regulates tumor microenvironment and reprograms immune cells to sustain tumor growth and progression.

## Introduction

Tumor angiogenesis, the formation of new blood vessels within a tumor, is one of the crucial steps of tumor growth and progression (Folkman, 1985). TECs are the cells lining the tumor-associated blood vessels (Dudley, 2012). Recent data suggest that TEC play a critical role in tumor growth and metastasis (Osawa et al., 2013; Yadav et al., 2015), and show characteristics of cancer stem cells (Dudley et al., 2008). TEC could regulate tumor microenvironment since they have direct contact with blood cells and vessel stromal cells. This regulation is mediated via production of increased levels of growth factors and cytokines, as well as via release of EV. EV are membrane vesicles containing a specific set of proteins, lipids, and nucleic acids, which could incorporate into recipient cells via cell surface receptors and regulate their gene expression (Quesenberry et al., 2015).

Extracellular vesicles are essential regulators of inflammation and angiogenesis (Baruah and Wary, 2019). Cancer requirement of inflammation is now widely described for almost all types of cancer since immune cells infiltrate tumor stroma and contribute to cancer development. EV secreted by tumor cells were shown to play an essential role in tumor niche establishment, metastasis (Hoshino et al., 2015), and regulation of immune reaction and inflammation (Ye et al., 2014; Grange et al., 2015). EV could activate immune cells by binding their surface receptors, such as MHC class I and II, CD47, TLR4, and others (Robbins et al., 2016).

Extracellular vesicles stimulate immune cells to express cytokines and growth factors critical for angiogenesis, TGF-β1, TNF-α, IL-6, and others (Roma-Rodrigues et al., 2014). Therefore, inflammation and angiogenesis are interdependent; moreover, those processes share participating immune cells and signaling pathways (Mamlouk and Wielockx, 2013; Szade et al., 2015).

Head and neck squamous cell carcinoma is the fifth most common cancer worldwide and has a high recurrence and metastasis rate (Argiris et al., 2008). It was shown that EV from HNSCC cells could inhibit T-cell proliferation and differentiation of pro-inflammatory cells Th1 and Th17 (Ye et al., 2014). HNSCC-derived EV are enriched in tumor antigens and potentially promote tumor progression (Principe et al., 2013). At present, limited information is available on the cellular and molecular functions of TEC from HNSCC and EV released by those cells (Rodriguez Zorrilla et al., 2019; Xie et al., 2019). Studying the TEC-EV molecular composition and specific functions in tumor development can define novel mechanisms of cancer immune editing, help developing novel therapeutic targets, and determine diagnostic signatures to be used for non-invasive liquid biopsies.

Here we investigated the role of EV released by TEC from HNSCC in the reprogramming of non-tumor cells, such as PBMCs or mesenchymal stem cells from adipose tissue (ASCs). Those cells underwent induction and contributed to tumor development by the secretion of growth factors and cytokines. This study characterized the regulation of the immune response by TEC-EV and showed the pro-tumorigenic functions of non-tumor cells after induction with TEC-EV.

## Materials and Methods

### Ethics Approval and Consent to Participate

Overall, we received the ethics approval for the isolation of human cells, such as TEC and tumor cells from HNSCC patients, as well as ASC from non-neoplastic patients. HNSCC tissues were isolated under approved protocol from the Ethics Committee of A.O.U. Città della Salute e della Scienza di Torino, Turin, Italy (CS2/1255 – Protocol number 0050416, May 16, 2019). ASC isolation was approved by the Ethics Committee of A.O.U. Città della Salute e della Scienza di Torino, Turin, Italy (CS/100 – Protocol number 12175, February 4, 2014). Adipose tissue was obtained from patients of non-neoplastic elective abdominal surgery. Informed consent was obtained from all patients according to the Helsinki Declaration. Laboratory researchers had no direct contact with the participants in the present study (patients’ anonymity was guaranteed).

### TEC Isolation

TEC were isolated from tumor tissue samples of HNSCC patients (stage IV). Specimens were finely minced and digested by incubation in DMEM medium with 220 U/mL collagenase I (Sigma) for 1 h at 37°C. After washings the cells in DMEM with 10% FBS (Lonza), endothelial cells were isolated from this filtered cells suspension using magnetic beads conjugated with anti-CD105 Ab (Supplementary Table S1) from magnetic cell-sorting MACS system (Miltenyi Biotech). TECs were grown in the complete EndoGro medium (Millipore) supplemented with 2% FBS, as previously described (Grange et al., 2006). Expression of CD31 by the isolated cells was confirmed by FACS analysis on the passages #1 and #4. TEC were used at 8–18 passages. TEC were functionally evaluated to form vessel-like structures at 4, 8, and 18 passages (see below).

Tumor cells were isolated from the specimens of four HNSCC male patients (mean age 62 ± 5) without sorting and cultured in MSCBM complete medium (Lonza). Tumor cells do not form vessel-like structures on Matrigel and have fibroblast-like morphology. The cells were allowed to undergo 8–18 passages before being used in the experiments.

### ASC Isolation

Adipose mesenchymal stem cells exhibit diverse inflammatory and secretory features depending on patient’ age, health, metabolism, and body size (Efimenko et al., 2014; Louwen et al., 2018). We used ASCs isolated from subcutaneous abdominal tissue of non-obese five male patients, submitted to non-neoplastic elective abdominal surgery (mean age 60 ± 8). Those adipose tissues were washed in sterile PBS and were minced into pieces of 2 mm in diameter. ASCs were isolated by enzyme digestion with 220 U/mL collagenase I type (Worthington Biochemical), and 40 U/mL dispase (Invitrogen Corporation) in DMEM, FBS deprived, and containing 100 units/ml penicillin, 0.1 mg/ml streptomycin, and 0.25 μg/ml amphotericin B (antibiotic/antimycotic solution, Sigma). The tissue was digested at 37°C for 30 min with constant mixing. Enzyme activity was neutralized with an equal volume of DMEM, containing 10% FBS. The digested suspension was centrifuged at 900 *g* for 10 min to obtain a high-density stromal vascular fraction pellet. The cell pellet was resuspended in MSCBM complete medium (Lonza) and cultured at 37°C in 5% CO_2_ incubator. After 2 days, the medium with detached cells was changed, and the adherent cells were cultivated until 100% confluence. ASC characterization was performed by FACS analysis for the positive expression of mesenchymal markers (CD105, CD73, CD90), and negative expression of hematopoietic markers (CD31) and by differentiation into adipogenic, osteogenic, and chondrogenic phenotypes as previously described (Kalinina et al., 2015). For our experiments, we used cells after 2–8 passages.

### PBMC Isolation

The fresh PBMC was isolated from 15 healthy donors. Their heparinized blood samples were used for the density gradient centrifugation. PBMC were seeded in 6 well plates at a density of 10 × 10^6^ cells per well in 2 ml of serum-free AIM V medium.

### TEC-EV Isolation

To isolate EV from TEC, TEC were washed with FBS-deprived DMEM and cultured in this medium for 18 h. The obtained conditioned medium was centrifuged for 30 min at 3000 *g* to remove cell debris and then filtered using 0.22 μm filters (MillexGP). The supernatants were then ultracentrifuged for 3 h at 100,000 *g* and 4°C using the Beckman Coulter Optima L-100K Ultracentrifuge with the rotor type 45 Ti 45000RPM. Minimum of 67 ml of conditioned medium was used for ultracentrifugation (maximum volume for the tubes). From this volume we extracted 0.5–2 × 10^11^ EV. The EV pellet was resuspended in DMEM supplemented with 1% of dimethyl sulfoxide (DMSO) then stored at −80°C until further use.

### ASC Stimulation With TEC-EV

To stimulate ASC with TEC-EV, we changed the complete growth medium of ASC culture to FBS-deprived DMEM. We added TEC-EV to the ASC culture to obtain the final concentration 10 × 10^3^ EV/cell. ASC were incubated with TEC-EV for 24 or 48 h to obtain ASCind. As a control, ASC were incubated with the equal volume of DMEM with 1% DMSO for 0, 24, or 48 h. After incubation, the cells were harvested, lysed by QIAzol Lysis Reagent and used for RNA isolation by RNAeasy kit (Qiagen), following manufacturer’s instructions.

### EV Isolation From Stimulated and Non-stimulated ASC

ASCind (stimulated for 24 h with TEC-EV) were used for isolation of their EV (ASCind-EV). Non-stimulated ASC were used for control EV isolation (ASC-EV). After 24 h-incubation with or without TEC-V, the growth medium of ASC was changed to FBS-deprived DMEM for the additional 24 h. We isolated EV from ASC conditioned medium as described for TEC-EV. From one ultracentrifuge tube (67 ml of conditioned medium) we extracted 1–5 × 10^10^ EV.

### PBMC Stimulation With EV

To stimulate PBMC with different EV (TEC-EV, ASC-EV, and ASCind-EV), we added EV to PBMC in the concentration of 1 × 10^3^ EV/cell for 48 h. After 48 h of incubation, the cultured PBMC and conditioned media were harvested for FACS, RT-PCR, and ELISA. For the T regulatory cell (Tregs) detection, PBMC were analyzed 5 days after incubation with EV.

### PBMC Consumption of EV

To demonstrate the intake of TEC-EV by PBMC, TEC-EV were labeled with PKH26GL green fluorescent cell linker (Sigma) dye for 30 min at 37°C and then washed and ultracentrifuged at 100,000 *g* for 1 h at 4°C. These labeled EV were added to PBMCs (1 × 10^3^ EV/cell) for different times (10, 30 min, 1, 3, or 24 h). The consumption of EV by PBMCs was evaluated by FACS (CytoFlex, Beckman Coulter).

### Vessel-Like Formation Assay

To verify the endothelial origin of TEC (donor cells, Table 1), or measure the angiogenesis (recipient cells II, Table 1), we have completed the vessel-like formation assay. For that, we seeded the cells onto Matrigel-coated wells in a 24-well plate (25 × 10^3^ cells per well), and cultured TEC in DMEM medium without FBS. In case of recipient cells II, 50% of the medium was replaced by the conditioned medium (CM) from the control PBMC or PBMCind. After incubation for 6 h, phase-contrast images (magnification, × 100) were recorded, and the total length of the network structures was measured using LAS software (Leica). In the case of recipient cells II, the total length per field was calculated in five random fields and expressed relative to CM PBMC control. Data were expressed as mean ± SEM.

**TABLE 1:** Scheme of the study.

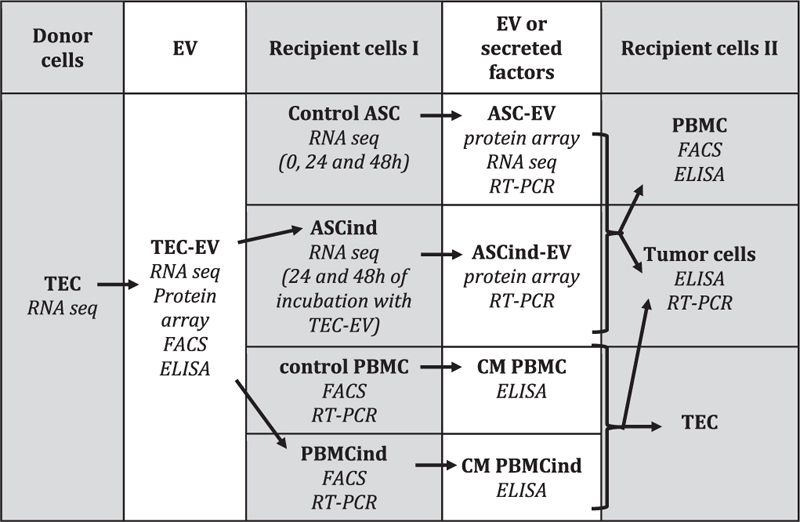

*In gray columns – cells used for the study, in white – EV (or conditioned media from PBMC). In bold – samples’ abbreviation used in text, in italic – methods for molecular composition analysis used for the sample.*

### Adhesion Assay

In this experiment, we measured PBMC or tumor cells adhesion on the endothelium. As a source of endothelial cells, we used HMEC (ATCC, PCS-110-010) cultured in EBM complete medium (Lonza). During the adhesion assay, all cell types (PBMC, endothelial cells, and tumor cells) were stimulated by the same EV to imitate the physiological conditions of EV circulation in the tumor blood vessels. HMEC seeded in 24-well plate were allowed to grow until 100% confluence. Those 100% confluent HMECs were stimulated with control ASC-EV or ASCind-EV in concentration 10 × 10^3^ EV/cell overnight. At the same time, PBMC or tumor cells were stimulated with the same EV (in the concentration 10 × 10^3^ EV/cell or 1 × 10^3^ EV/cell for tumor cells or PBMC respectively). Next day, tumor cells and PBMC were labeled with PKH26GL red fluorescent cell linker (Sigma) according the manufacturer’s instructions. Labeled PBMC (2 × 10^5^ cells) or tumor cells (1 × 10^3^) resuspended in 0.5 ml of EBM complete medium were added to each well of confluent and stimulated HMEC for 1 h incubation at 37°C. The non-adherent cells were removed by supernatant aspiration and two additional washing steps with PBS. Adherent cells were counted by digital analysis (Leica application suite V4.9). Four independent experiments were performed in duplicates; the results are expressed as mean of cells counted in 10 fields. The non-stimulated cells (HMEC, PBMC, or tumor cells) were used as negative controls.

### Tumor Cell Proliferation

The proliferation of tumor cells was measured by BrdU incorporation using Cell Proliferation ELISA BrdU (colorimetric) kit (Roche, #11647229001) according to the manufacturer’s instructions. Tumor cells were cultured in DMEM deprived FBS and were stimulated by 50% diluted conditioned medium from control PBMC or PBMCind. Tumor cells were stimulated with such medium for 72 h.

### Tumor Cell Invasion

The effect of ASC-EV on tumor cell invasion was detected by the Transwell assay (COSTAR transwell, Corning Incorporated). Tumor cells were resuspended in DMEM (serum-free) and seeded into the upper compartment of an invasion chamber (50 × 10^3^ cells per well) containing a polycarbonate membrane with an 8 μm pore size, which was coated with a layer Matrigel (MatrigelTM, Becton Dickinson). ASC-EV or ASCind-EV were added to the cells into the concentration 5 × 10^8^ EV/well. DMEM with 10% of FBS was used as the attractant and added to the bottom well. After 24 h of incubation, the invasive cells migrated through the Matrigel to the bottom side of the membrane. The migrated cells were stained with Mayer dye and counted. Every experiment was repeated six times.

### RNA Sequencing and Analysis of TEC-EV

Four TEC-EV samples were collected from the same TEC culture on different cell passages and were used for RNA sequencing using Lexogen^1^ service (Vienna, Austria). From 1 × 10^11^ of EV was extracted around 30 μg of RNA. The service included the comparison of RNA carried by TEC-EV with RNA carried by ASC-EV by DeSeq2. The top 1000 up-regulated genes in TEC-EV relative to ASC-EV (p_adj_ < 0.1) were used in the Panther classification system^2^ for functional clustering.

### RNA Sequencing of ASC Stimulated With TEC-EV

RNA from treated and non-treated cells was extracted using the mirVana miRNA Isolation Kit (Ambion, Forster City, CA, United States). RNA sequencing was performed, as previously described (Guo et al., 2017). Briefly, samples were required to achieve an RNA Integrity Number (RIN) of at least 7.0. A stranded RNA library was prepared using the Illumina TruSeq stranded total RNA seq poly A + Gold kit (San Diego, CA, United States), and then ribosomal RNA reduction was performed from 400 ng of total RNA and purified with AMPure XP magnetic beads.

Sequencing was performed using the HiSeq 2500 platform sequencer (Illumina) and the TruSeq Cluster Kit, resulting in approximately 80 million paired reads per sample. Next, the RNA sequencing data were normalized based on the version 2 protocols developed by TCGA (Cancer Genome Atlas Network, 2015). The alignment was performed using MapSplice2 version 2.0.1.9 (Wang et al., 2010) to the GRCh37/hg19 genome assembly. Gene expression values were quantified from RNA sequencing data using RSEM version 1.2.9 (Li and Dewey, 2011) and upper quantile normalized. We found patterns on this data by performing CoGAPS analysis on log transformed, upper quantile normalized data (Fertig et al., 2010). This analysis identified five patterns in the dataset, which were associated with biological pathways by applying the CoGAPS gene set statistic to Hallmark pathways (Liberzon et al., 2015) in MSigDB version 5.2. CoGAPS patterns are visualized by creating a heatmap of the gene expression values for patternMarker genes that are uniquely associated with each process (Stein-O’Brien et al., 2018).

### Protein Array of EV

Protein composition of EV was identified by AAH-BGL-100004 Human L1000, Glass Slide array (RayBiotech). To extract protein peptides, EV were lysed by 2x Cell Lysis Buffer (RayBiotech, Inc.) and ultrasonicated 30 s at 4°C (Microson Ultrasonic cell disruptor; 47 kHz frequency and 185 Watt peak power). The protein concentration was measured by BCA Protein Assay Kit (Thermo Fisher). One μg of total protein was used for the protein array according to manufacturer instructions. This array provides detection of 1000 secreted proteins. For each set of EV, we performed two technical repeats. Only proteins, consistently detected in both replicates were used for functional clustering by Panther classification system^2^.

### Real-Time PCR Analysis

Total RNA from stimulated and non-stimulated PBMC, and from EV released by ASC controls and ASCind, was extracted using RNAeasy kit (Qiagen). Approximately 200 ng of RNA was reverse transcribed into complementary DNA (cDNA) using miScript II RT Kit (Qiagen) according to manufacturer’s protocol.

The analysis of the TGF-β1 and MALAT1 RNA expression, as well as the expression of endogenous controls, ACTB and 18S RNA, was performed using miScript SYBR Green PCR kit (Qiagen). Specific primers for RT-PCR (TGF-β1 forward CTAATGGTGGAAACCCACAACG, reverse TATCGCCAGGAATTGTTGCTG; MALAT1 forw ard CCCCTGGGCTTCTCTTAACA, reverse GCTAGATCAAA AGGCACGGG; ACTB forward CATGTACGTTGCTATCCA GGC, reverse CTCCTTAATGTCACGCACGAT, 18S RNA forward AGAAACGGCTACCACATCCA, reverse CCC TCCAATGGATCCTCGTT) were obtained from primer bank^3^. Used real-time cycler conditions were following: PCR initial activation step 15 min 95°C; 40 steps of Denaturation (94°C, 15 s), Annealing (60°C, 15 s), Extension (72°C, 15 s); fluorescence data collection after Extention of every cycle; melting curve data between 60 and 95°C. The RT-PCR was performed on the QuantStudio 12K Flex real-time PCR instrument (Applied Biosystems). The differential expression analysis was performed using Expression Suite Software (Thermo Fisher), using endogenous controls.

### Fluorescence-Activated Cell Sorting

Fluorescence-activated cell sorting was used to define surface protein markers on cells and EV. A pool of approximately 5 × 10^10^ cells or EV dissolved in 100 μl was incubated with the particular antibody (Supplementary Table S1) for 30 min at room temperature. Then the final volume was increased until 300 μl and FACS analysis was performed using CytoFlex from Beckman Coulter. To detect EV we used Violet Side Scatter (VSSC) that permit to detect even smaller vesicles, as the refraction index of EVs is inversely proportional to the size of the vesicle. We were able to select the population of EV using CD63, CD81, and Annexin V as positive controls. The median fluorescence intensity (MFI) was corrected for background and gated based on their respective fluorescence intensity as per manufacturer’s instructions. The FITC, PerCP, or PE non-immune isotypic IgG were used as a negative control.

### ELISA Assay

ELISA assay was performed using DuoSet^®^ ELISA Development Systems (R&D Systems) according to manufacturer instructions. Briefly, the suspension of EV or cells was mixed with RIPA buffer and ultrasonicated. Then, 30 μg of total protein was used for ELISA. TEC-EV were analyzed using kits specific to IGF1, IL-1β, TGF-β1, and VEGF. PBMC or tumor cells were analyzed using kits specific to IFN-γ, IL-6, IL-10, TGF-β1, TNF-α, and VEGF.

### Statistical Analysis

All reported *p*-values were calculated using pair-wise Student’s *t*-test, comparing a condition of interest with its corresponding control. *p*-Value < 0.05 was considered as significant.

## Results

### The Study Flow

We performed two rounds of EV collection and used them for stimulation of the recipient cells, as described in Table 1. The study was started from the isolation of the TEC (donor cells) and TEC-EV collection and characterization. TEC-EV were analyzed by RNA sequencing, protein array, FACS, and ELISA and were used for stimulation of two types of recipient cells I (ASCs and PBMC). ASCs induced with TEC-EV (ASCind) were analyzed by RNA sequencing at 24 and 48 h after the stimulation. Non-stimulated control ASCs and TEC were also analyzed by RNA sequencing for comparative analysis. PBMC stimulated with TEC-EV (PBMCind) were analyzed by ELISA and FACS to demonstrate activation and differentiation. At the second round, control ASCs or ASCind were used for EV collection (ASC-EV or ASCind-EV), which were used for stimulation of the recipient cells II (tumor cells or PBMC).

### EV Characterization

TEC-EV, ASC-EV, and ASCind-EV were analyzed using Nanoparticle Tracking Analysis (Malvern Instruments, Ltd.) and transmission electron microscopy. Mean size of EV evaluated by electron microscopy was 90 nm (± 20); we did not detected vesicles bigger than 100 nm o smaller than 30 nm. According to Western blot all types of EV expressed CD63 and CD81 exosome markers but not endoplasmic reticulum calnexin (Supplementary Figure S1).

### Protein and RNA Composition of TEC-EV

**FIGURE 1:**
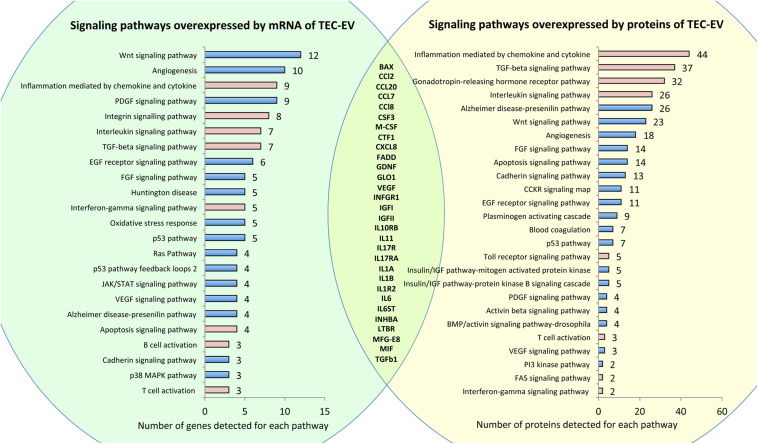
RNA and protein composition of TEC-EV. Left panel (in green) shows pathways overrepresented by mRNAs detected by RNA sequencing of TEC-EV. Right panel (in yellow) shows pathways overrepresented by proteins, detected by protein array in TEC-EV. Pathways relevant to inflammation are marked in pink. In the intersection, there is a list of genes present in TEC-EV at both mRNA and protein levels.

To study the molecular composition of TEC-EV, we ran RNA sequencing and protein array on TEC-EV. Analysis of RNA sequencing of TEC-EV revealed that TEC-EV contained mRNA from inflammation, interleukin signaling, TGF-β1, and T and B cell activation pathways (Figure 1, left). According to RNA sequencing data TEC-EV are enriched with TGF-β1 mRNA and long non-coding RNA MALAT1. TGF-β1 is a mediator of immunosuppression and Treg differentiation (Taylor et al., 2006; Konkel et al., 2017), while the MALAT1 was described as a downstream mediator of TGF-β1 effect in immunosuppression (Yang et al., 2016; Masoumi et al., 2019).

Analysis of TEC-EV proteins showed enrichment of similar signaling pathways relevant to inflammation (Figure 1, right). Comparison of gene lists, revealed by RNA sequencing and by protein array, confirmed that TEC-EV carry mRNA and proteins of genes relevant to inflammation (Figure 1, intersection of the circles), such as interleukins and their receptors IL-1a/b, IL1R2, IL6, IL10RB, IL11, IL6ST, IL13RA1, IL17R, IL7RA, IL17RA, chemokines CCL2, CCL7, CCL8, CXCL8, CCL20, growth factors TGF-a, TGF-β1, GDNF, VEGFa, M-CSF, CSF3, IGFI, IGFII, CTF1, and others (MFG-E8, BAX, FADD, GLO1, INHBA, IFNA/BR1, IFNGR1, and MIF). Using ELISA, we further confirmed the enrichment of TEC-EV with TGF-β1, IL-1β, IGF1, and VEGF.

Fluorescence-activated cell sorting analysis of TEC-EV showed the surface representation of HLA class I, HLA G, CD47, TLR4, VE-cadherin, CD44, CD63, CD81, ICAM, VCAM, and CD31 proteins. Interestingly, TEC-EV were negative for CD45, CD279, CD274, and HLA-DR (Supplementary Figure S2).

The molecular composition of TEC-EV suggests that they are enriched with transcripts and proteins of different immune pathways, and therefore can potentially regulate immune reaction.

### TEC-EV Activated PBMC and Stimulated T Regulatory Cell Formation

**FIGURE 2:**
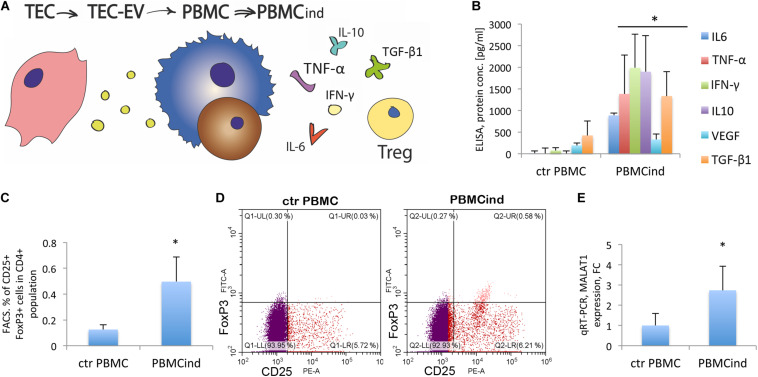
PBMC stimulation with TEC-EV. **(A)** Scheme of the experiment: TEC- EV were isolated from TEC and used for the induction of PBMC (PBMCind). After that, PBMCind and control non-stimulated PBMC (ctr PBMC) were analyzed by FACS, PCR, and ELISA. **(B)** Diagram of the PBMC cytokine secretion: IL-6, IFN-γ, TNF-α, and IL-10, as well as TGF-β1 and VEGF are secreted significantly more by PBMCind than control PBMC. **(C)** Diagram of Treg formation by PBMCind. **(D)** Representative FACS dot-plots of double-positive FoxP3^+^ (FITC) and CD25^+^ (PE) cells (up-right quarter, Q2), selected from CD4^+^ cells. **(E)** Diagram of MALAT1 expression in control PBMC and PBMCind. Data are represented as mean (SD), ^∗^*p* < 0.05 vs. PBMC, *n* = 15.

To study the direct effect of TEC-EV on immune cells, we stimulated PBMC (Table 1, recipient cell I, Figure 2A) with the EV in serum-free medium, and analyzed the changes in proliferation, differentiation, and cytokine expression.

First, we demonstrated the uptake of TEC-EV by PBMC. We added TEC-EV, labeled with PKH67GL green fluorescent dye, to freshly isolated PBMC and performed FACS analysis at different time points. TEC-EV were absorbed faster by monocytes since after 3 h more than 70% of these cells were fluorescent, while lymphocytes even after 24 h were < 40% positive (Supplementary Figure S3).

Interestingly, when we isolated monocytes and lymphocytes from the total PBMC population to perform a separated stimulation with TEC-EV, TEC-EV were hardly able to support the viability of separated cells in serum-free medium, and we did not see any changes in proliferation and cytokine secretion. Therefore, we performed all following experiments on total PBMC population.

Using FACS analysis, we compared the PBMC induced with TEC-EV (PBMCind) with its unstimulated control (ctrPBMC). The analysis showed that TEC-EV activated monocytes, according to enhanced expression of CD14 and CD69 (Supplementary Figure S4).

TEC-EV increased the expression of CD25, the marker of proliferation, in both monocytes and lymphocytes (fold change 3.5 for monocyte fraction, and 1.6 for lymphocytes, *p* < 0.05, Supplementary Figure S4). These data suggest that TEC-EV stimulated the proliferation of both cell types in PBMC.

We have also evaluated the role of TEC-EV on the differentiation of the PBMC cells. Thus, TEC-EV increased the expression of CD163 (fold change 2.6, *p* = 0.03) and CD206 (fold change 4.3, *p* = 0.05) in monocyte fraction. These data suggest that TEC-EV could induce differentiation of monocytes into immunosuppressive macrophages type M2. Also, TEC-EV significantly increased the expression of CD274 (PD-L1) in monocyte fraction (fold change 4.4, *p* = 0.05, Supplementary Figure S4).

ELISA analysis of cytokine expression in PBMCind showed a significant increase of IFN-γ, TNF-α, IL-6, VEGF, IL-10, and TGF-β1 expression relative to unstimulated controls (Figure 2B). TGF-β1 plays an essential role in u formation. Indeed, TEC-EV stimulation significantly increased the population of Tregs (CD4^+^CD25^+^FoxP3^+^) in PBMCind (fold change 3.96, *p* = 0.049) (Figures 2C,D). We also showed that PBMC treatment with TEC-EV increased the expression of MALAT1 in PBMCind (fold change 2.7, *p* = 0.04, Figure 2E). Its increased expression could be relevant for both M2-type differentiation of macrophages and Treg formation (Masoumi et al., 2019). All of these results suggest that TEC-EV induce an immunosuppressive phenotype (formation of Treg and CD206^+^, CD163^+^ monocytes) in PBMC via influence on monocytes and lymphocyte proliferation and differentiation.

### PBMC Induced With TEC-EV Stimulate Tumor Cell Proliferation and Angiogenesis *in vitro*

**FIGURE 3:**
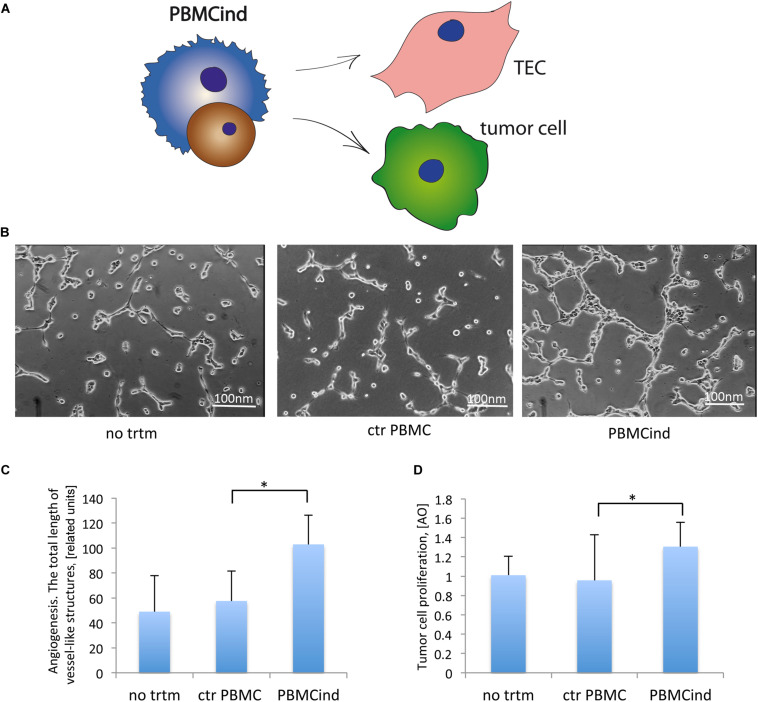
PBMCind regulated tumor cell growth and function. **(A)** Scheme of the experiment: conditioned medium from control PBMC and PBMCind were used to stimulate TEC or tumor cells, then angiogenesis *in vitro* and proliferation were measured. As negative control were used TEC or tumor cells without any treatment (no trtm). **(B)** Representative images of angiogenesis *in vitro* by TEC, stimulated with conditioned medium obtained from control PBMC or PBMCind. **(C)** Diagram of the total length of vessel-like structures formed *in vitro* by TEC, stimulated with conditioned medium from control PBMC or PBMCind. **(D)** Diagram of stromal cell proliferation. Data are represented as mean (SD), ^∗^*p* < 0.05 vs. CM PBMC CONTROL, *n* = 6.

To demonstrate the functional activity of the PBMC induced with TEC-EV, we used the conditioned medium from PBMCind for stimulation of tumor cells and TEC (Table 1, recipient cells II, Figure 3A).

Conditioned medium from PBMCind enhanced the pro-angiogenic properties of TEC *in vitro* (fold change 1.8, *p* = 0.001 Figures 3B,C). That also correlated with the increased production of VEGF, IL-6, and TGF-β1, the effectors of tumor angiogenesis (Figure 2B). Moreover, conditioned medium from PBMCind stimulated the proliferation of tumor cells (fold change 1.4, *p* = 0.04, Figure 3D).

According to these results, we suggested that stimulated PBMC stimulated the growth of tumor mass and its angiogenesis.

### TEC-EV Regulate Gene Expression in ASCs

**FIGURE 4:**
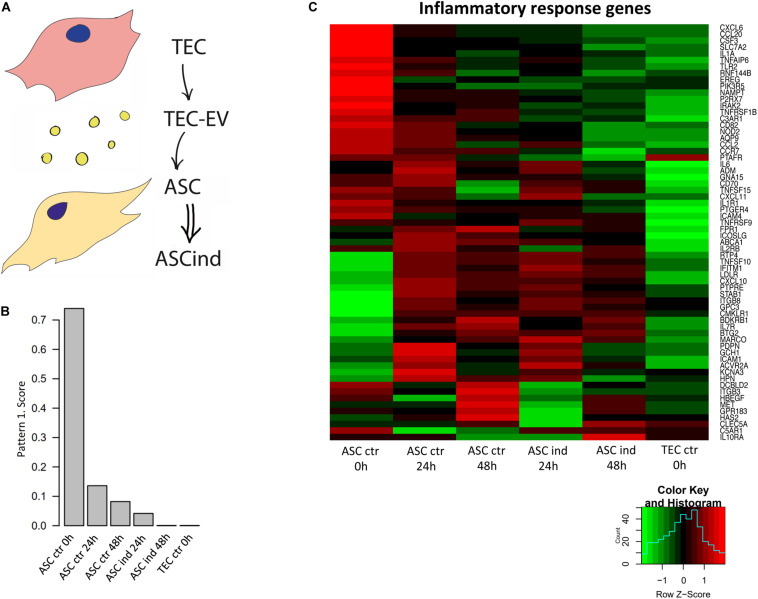
TEC-EV influence on ASCs. **(A)** Scheme of the experiment: TEC-EV were used for the induction of ASC (ASCind). **(B)** Diagram of the relative expression of genes from pattern 1, obtained after analysis of RNA sequencing data of ASCs stimulated with TEC-EV during 24 and 48 h. **(C)** Gene expression heatmap of the inflammatory response group. ASCs at time point 0 h were taken as control (ASC ctr 0 h), also TEC were taken as control cells (TEC ctr). ASCs, induced during 24 and 48 h (ASCind 24 h and ASCind 48 h) were analyzed in parallel with non-stimulated ASCs at the same time points (ASC ctr 24 and ASC ctr 48 h respectively).

To study if TEC-EV induce immunosuppressive phenotype in stromal cells, we analyzed the effect of TEC-EV on gene regulation in ASCs directly (Table 1, recipient cells I), since adipose tissue is present in close proximity of primary head and neck tumors. For this reason, we induced ASCs with TEC-EV (ASCind) for 24 and 48 h. We compared ASCind and its unstimulated controls with the donor TEC cells. The gene expression in each experiment was analyzed by RNA sequencing (Figure 4) and was used to define gene expression signatures of acquired cancerization of ACS by the Bayesian matrix factorization algorithm (CoGAPS) (Fertig et al., 2010).

The CoGAPS analysis demonstrated the coherent down-regulation of a set of genes after the exposure of ACS to TEC-EV (Figures 4B,C). Such downregulation correlated with the overall nature of HNSCC, known for its tumor suppressor-driven biology. This pattern contains genes that are highly expressed in control ASCs, not expressed in TEC, and down-regulated in ASCs after TEC-EV stimulation (Figure 4B). These genes play a role in pathways involved in tumor immune response (INFLAMMATORY_RESPONSE, COMPLEMENT, IL6_JAK_STAT3_SIGNALING) and in fatty acid metabolism pathway (FATTY_ACID_METABOLISM). These data suggest immunosuppression and transdifferentiation of ASC after TEC-EV induction.

### ASCind-EV Activated PBMC and Stimulated Tregs Formation

**FIGURE 5:**
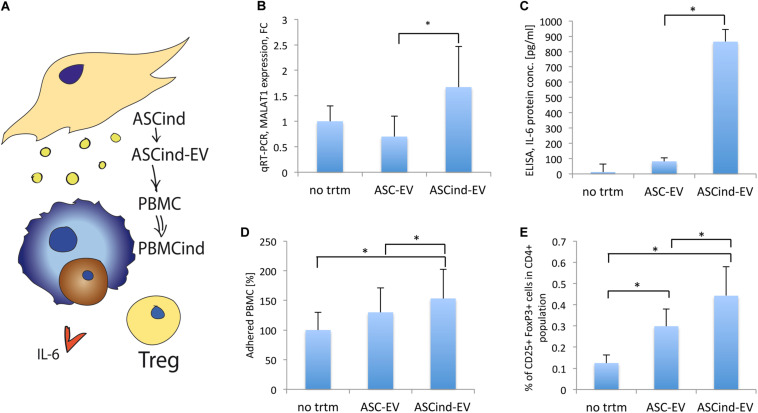
The influence of the ASCind on PBMC activity. **(A)** Scheme of the experiment: ASCind-EV were used for stimulation of PBMC, after that ELISA and FACS analysis of the stimulated PBMC were performed. **(B)** Diagram of MALAT1 expression in PBMC stimulated with control ASC-EV or ASCind-EV respect to non-treated PBMC (mean (SD), ^∗^*p* < 0.05 vs. ASC-EV, *n* = 8). As negative control were used PBMC without any treatment (no trtm). **(C)** Diagram of IL-6 secretion by PBMC, stimulated or not with ASC-EV and ASCind-EV [mean (SD), ^∗^*p* < 0.05 vs. ASC-EV, *n* = 8]. **(D)** Diagram of the PBMC adhesion on endothelium after stimulation with ASC-EV or ASCind-EV [mean (SD), ^∗^*p* < 0.05 vs. ASC-EV, *n* = 8]. **(E)** Diagram of Treg formation by PBMC, stimulated with ASC-EV or ASCind-EV [mean (SD), ^∗^*p* < 0.05, *n* = 6].

To demonstrate if tumorigenic induction could be passed from TEC through ASCs to secondary cells by EV, we collected EV released by ASCind (ASCind-EV) or control ASCs (ASC-EV) and used these EV for stimulation of the recipient cells II (PBMC) (Table 1, recipient cells II, Figure 5A).

The ASCind-EV and ASC-EV were characterized by protein array. The protein level of CXCL2, CXCL5, FGF2, PGF, MMP1, MMP3, CCL2, CCL7, S100A12, PRL, NID1, IL-10, TGF-α, and TGF-β1-5 was increased in ASCind-EV relative to control ASC-EV (Supplementary Figure S5A). Many of those proteins are involved in tumor growth.

ASCind-EV had an increased level of lncRNA MALAT1 (Supplementary Figure S5B). ASCind-EV significantly increased the secretion of IL-6 by and expression of MALAT1 in PBMC (Figures 5B,C). This strongly correlated with the increased expression of both IL-6 and MALAT1 in PBMC after TEC-EV stimulation (Figures 2B,E).

Functionally, PBMC adhesion on endothelium increased after the stimulation with ASCind-EV (Figure 5D). Moreover, ASCind-EV increase the formation of Treg relative to control ASC-EV (Figure 5E and Supplementary Figure S6).

These findings suggest that ASC once stimulated with TEC-EV could induce an immunosuppressive phenotype of PBMC through the released ASCind-EV.

### ASCind-EV Stimulate Tumor Cells

**FIGURE 6:**
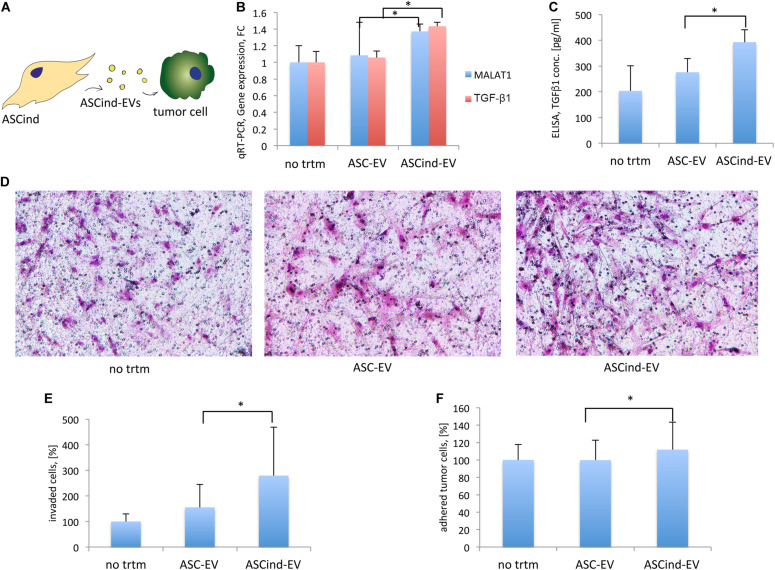
The influence of the ASCind on tumor cell activity and functions. **(A)** Scheme of the experiment: ASCind-EV were used for stimulation of tumor cells, after that tumor cell gene expression and secretory activity, adhesion, and invasion were measured. **(B)** Diagram of the MALAT1 and TGF-β1 gene expression in tumor cells stimulated with ASC-EV or ASCind-EV [mean (SD), ^∗^*p* < 0.05, *n* = 6]. As negative control were used tumor cells without any treatment (no trtm). **(C)** Diagram of TGF-β1 secretion by tumor cells stimulated with ASC-EV or ASCind-EV [mean (SD), ^∗^*p* < 0.05 vs. ASC-EV, *n* = 6]. **(D)** Representative images on tumor cell invasion. Ability to invade into Matrigel was quantified by counting the number of invaded cells under a phase-contrast microscope. **(E)** Diagram on tumor cell invasion. **(F)** Diagram of the tumor cell adhesion on endothelium [mean (SD), ^∗^*p* < 0.05 vs. ASC-EV, *n* = 6].

To study the effect of ASCind-EV on tumor stroma, we stimulated tumor cells with the ASC-EV or ASCind-EV and analyzed their biological activity (Table 1, recipient cells II, Figure 6A). Tumor cell stimulation with ASCind-EV led to a significant increase of TGF-β1 expression and secretion (Figures 6B,C). Also, the expression of MALAT1 was significantly enhanced in tumor cells after the stimulation with ASCind-EV (Figure 6B). This strongly correlates with the increased expression of both TGF-β1 and MALAT1 in ASCind (Supplementary Figure S5).

Tumor cell invasion and adhesion on endothelium (Figures 6D–F) were also increased, suggesting the increased capacity of tumor cells to invade from circulation to tissue after being stimulated with ASCind-EV.

These findings suggest that ASC once stimulated with TEC-EV induce the migration of tumor cells from the bloodstream that could be relevant to subsequent metastasis.

## Discussion

In our study, we showed that TEC-EV could transmit their oncogenic signals, and this process represents a contagious carcinogenesis. Unlike the metastatic spread of tumor cells, this mechanism regulates tumor microenvironment and reprograms immune cells to sustain tumor growth and progression. In the present study for the first time, we showed the pro-tumorigenic functions of the surrounding non-tumor cells. Such data correlates with previous knowledge that cancerous EV reprogram the immune and stromal cells of the microenvironment to enhance tumor growth, angiogenesis, migration, and tumor tolerance by immune cells (Grange et al., 2015; Hoshino et al., 2015).

Extracellular vesicles, released from tumor cells, including breast cancer (Lombardo et al., 2018), renal carcinoma (Lopatina et al., 2019b), leukemia (Paggetti et al., 2015) and others (Guo et al., 2019), participate in the formation of tumor-promoting pre-metastatic niches (Hoshino et al., 2015). Unfortunately, similar studies for HNSCC are limited. In this work, we explored the role of cancer-derived EV on the local microenvironment of HNSCC tissues and the mechanism of spread of the pro-oncogenic signals. We have shown that this effect is achieved through stromal and immune cells.

A short-time (24 h) stimulation of the recipient cells I (PBMC or ASCs) with TEC-EV changed the gene expression of these cells into pro-oncogenic direction. Thus, TEC-EV regulate gene expression of ASC and decrease their immune reaction. On the other hand, TEC-EV also activate PBMC, regulate cytokine secretion, stimulate Treg cell formation, and activate immunosuppression. Moreover, PBMC can get further immunomodulated by EV from the induced ASCs. Therefore, TEC acting through TEC-EV have both direct and indirect effect on the immune system. This non-metastatic spread of tumor signals to the healthy surrounding cells leads to the carcinogenesis. Furthermore, stimulated PBMC and ASC can carry out the induced pro-oncogenic signals to tumor tissues, enhancing proliferation, adhesion, and invasion of tumor cells, and angiogenesis of TEC. This process makes tumor-surrounding immune and adipose cells function in favor of tumor development and amplify the pro-oncogenic stimulus.

The immune system plays an important role in tumor development. We have shown that EV are get absorbed mainly by monocytes, as well as by lymphocytes. These data is strongly supported by the known internalization of EV by macrophages (Imai et al., 2015). Moreover, our data strongly correlated with the described role of different stromal non-tumor cells, such as endothelial cells (Yu et al., 2010), fibroblasts (Knuchel et al., 2015), mesenchymal stem cells (Lee and Hong, 2017), adipose-derived stem cells (Wei et al., 2015), in the regulation of growth and progression of tumors.

Obesity is considered to be another risk factor for cancer progression (Abd Elmageed et al., 2014). Chronic inflammation links obesity and tumor development, sharing plenty of molecular mechanisms. Recent studies have shown that ASCs can be easily induced into tumor direction (Razmkhah et al., 2018). Moreover, HNSCC is often laid within close proximity of adipose tissue (Tan et al., 2015; Gama et al., 2017). We presume that the inflammatory status and biodiversity of each sample of ASC did not affect the results of our study, since we demonstrated the immunosuppressive effect of ASCind-EV in comparison with control ASC-EV, collected from the same ASC culture.

The immunosuppressive function of EV during cancer formation was recently reported for head and neck cancer development (Ludwig et al., 2017). Thus, EV from the plasma of patients with the stages III/IV HNSCCs exhibit active immune suppression functions through the synthesis of adenosine and stimulation of Treg (Maybruck et al., 2017; Theodoraki et al., 2018), by inducing apoptosis of CD8^+^ T cells and suppression of CD4^+^ T-cell proliferation (Ludwig et al., 2017), by inducing immunosuppressive phenotype of CD8^+^ cells (Maybruck et al., 2017) and carrying immunosuppressive proteins, such as PD-1, PD-L1, Fas, FasL, CTLA-4, TRAIL, CD73, COX2, TGFβ-LAP (Ludwig et al., 2017). These data can explain a high speed of HNSCC cancer formation and its high rate of cancer recurrence. The recurrence rate for HNSCC is 25–48%, and sometimes up to 67% and especially higher for the late-stages III–IV tumors (Schwartz et al., 2000).

We delineated the genes expression signatures for the acquired cancerization of ASCs, by the CoGAPS [Coordinated Gene Activity in Pattern Sets, algorithm (Fertig et al., 2010)]. This algorithm was applied to the gene expression time-course data of ASC stimulated with TEC-EV. CoGAPS was previously used to successfully delineate the time-course data of therapeutic response in breast and HNSCC (Hill et al., 2016; Stein-O’Brien et al., 2018). In our case, it detected the critical role of inflammatory response gene set to respond to TEC-EV stimuli within 24 h (Figure 4).

Tumor and adjacent non-tumor cells in its microenvironment, consisting of heterogenic malignant, stromal, and immune cells, are found in continuous reciprocal communication. The induction of non-tumor cells toward the oncogenic direction by squamous tumor cells was first described as field cancerization in 1953 (Slaughter et al., 1953). From that time, the gene expression reciprocal regulation of the adjacent tumor and non-tumor cells was demonstrated to almost all types of cancer (Curtius et al., 2018). The recent single-cell analysis of HNSCC showed that stromal and immune cells had consistent gene expression across patients, whereas malignant cells varied their expression signatures (Puram et al., 2017). We showed how TEC could regulate gene expression of PBMC and ASC through EV, making them favor tumor development. In particular, we showed that TEC-EV increased expression of TGF-β1 and MALAT1 in target cells. According to the published data, TGF-β1 is involved not only in immune suppression in tumor microenvironment (Takahashi et al., 2017), but also promotes heterogeneity and cancer stem cell formation (Oshimori et al., 2015) and epithelial-mesenchymal transition (Horiguchi et al., 2012), supporting our findings. Therefore, TEC-EV stimulated target cells to secret TGF-β1 necessary for tumor cell differentiation and immune surveillance escape.

The higher expression of MALAT1 was detected in PBMC and in ASC-EV after direct stimulation with TEC-EV, as well as in PBMC and tumor cells after stimulation with ASCind-EV. MALAT1 was shown as a circulating biomarker of head and neck cancer (He et al., 2017). TGF-β1 could activate the transcription of MALAT1 (Wang et al., 2018), and MALAT1 in its turn could activate the transcription of LTBP3, a gene that regulates the bioavailability of TGF-β (Li et al., 2014). We have shown that TEC-EV could enhance the secretion of both MALAT1 and TGF-β1 within ASCind-EV, and that ASCind-EV could induce their expression by tumor cells, play a role in Treg formation, and stimulate their immunosuppressive activity. This transfer of MALAT1 could be one of the possible molecular mechanisms that regulate tumor microenvironment. Future studies are needed to understand those mechanisms.

*In vitro* we have shown the immunosuppressive effects of EV derived from TEC of HNSCC, and transfer of these effects through non-tumor cells, suggesting, that TEC-EV can reprogram non-tumor cells into an oncogenic phenotype in short time. These data need the *in vivo* confirmation, and we plan to investigate the influence of TEC-EV on tumor growth and tumor niche formation using laboratory *in vivo* models (Grange et al., 2011). Such prospective work will also help us to define the role of TEC-EV on the CoGAPS inflammatory cell pattern in mice. Since EV, in general, have complex structures with highly specific and diversified molecular composition, it is challenging to identify the molecular mechanism of its action. We are going to down-regulate the MALAT1 expression in TEC to see the molecular changes in TEC-EV and their effect on Treg formation and TGF-β1 production by target PBMC.

Overall, our data suggest that tumor tissues trigger many self-promoting mechanisms that amplify the pro-oncogenic signals, with a specific role of TEC, which line tumor blood vessels, and their EV in this process. Moreover, our data suggest that TEC might be essential targets for the development of cancer therapy.

## Data Availability Statement

The datasets presented in this study can be found in online repositories. The names of the repository/repositories and accession number(s) can be found in the article/ Supplementary Material.

## Ethics Statement

The studies involving human participants were reviewed and approved by Ethics Committee of A.O.U. Città della Salute e della Scienza di Torino. The patients/participants provided their written informed consent to participate in this study.

## Author Contributions

TL and DG: conception and design. TL, EF, EJF, and LK: development of methodology. LK, EJF, BB, RR, RA, and GP: acquisition of data (acquired and managed patients, provided facilities, etc.). TL, EJF, AF, LD, and DG: analysis and interpretation of data (e.g., statistical analysis, biostatistics, computational analysis). TL, LD, AF, MB, GC, and DG: writing, review, and/or revision of the manuscript. MB and GC: administrative, technical, or material support (i.e., reporting or organizing data, constructing databases). All authors contributed to the article and approved the submitted version.

## Conflict of Interest

The authors declare that the research was conducted in the absence of any commercial or financial relationships that could be construed as a potential conflict of interest.

## Footnotes

1 www.lexogen.com

2 http://www.pantherdb.org

3 https://pga.mgh.harvard.edu/primerbank/

## Abbreviations

ACTB: actin beta 1
ASCs: adipose stem cells
CD14: cluster of differentiation 14
CD31: cluster of differentiation 31
CD47: cluster of differentiation 47
CD73: cluster of differentiation 73
CD90: cluster of differentiation 90
CD105: cluster of differentiation 105
CD163: cluster of differentiation 163
CD206: cluster of differentiation 206
CM: conditioned medium
Ctr: control
DMEM: Dulbecco’s modified Eagle medium
DMSO: dimethyl sulfoxide
ELISA: enzyme-linked immunosorbent assay
EVs: extracellular vesicles
FACS: fluorescence-activated cell sorting
FBS: fetal bovine serum
HLA: human leukocyte antigen
HMEC: human dermal microvascular endothelium
HNSCC: head and neck squamous cell carcinoma
IFN- γ: interferon gamma
IL-6: interleukin 6
IL-10: interleukin 10
lncRNA: long non-coding RNA
MALAT1: metastasis associated lung adenocarcinoma transcript 1
MHC: major histocompatibility complex
PBMC: peripheral blood mononuclear cell
PBS: phosphate-buffered saline
RNA: ribonucleic acid
RT-PCR: reverse transcription polymerase chain reaction
TECs: tumor endothelial cells
TGF- β 1: transforming growth factor beta-1
Th1: T helper type 1
Th17: T helper type 17
TLR4: toll-like receptor 4
TNF- α: tumor necrosis factor alpha
Treg: T regulatory cells
Trtm: treatment
VEGF: vascular endothelial growth factor.
