# Supplementary material for: Extracellular Vesicles Released by Tumor Endothelial Cells Spread Immunosuppressive and Transforming Signals Through Various Recipient Cells
Source: Front Cell Dev Biol. 2020 Sep 9;8:698. doi: 10.3389/fcell.2020.00698 (PMC7509153; doi:10.3389/fcell.2020.00698)
Supplement: Supplementary file 1 [file Table_1.docx]

Supplement Table 1. Antibodies used for FACS of TEC-EVs and PBMC.

| Protein name | Catalog # | Company | Conjugated  fluorochrome | What experiment | Marker of/ function |
| --- | --- | --- | --- | --- | --- |
| CD73 | 550257 | BD Pharmigen | PE | ASC characterization | Mesenchymal stem cells |
| CD90 | 555595 | BD Pharmigen | FITC | ASC characterization | Mesenchymal stem cells |
| CD105 | 130-051-201 | Miltenyi | microbeads | TEC isolation | Endothelial cells |
| CD63 | 557288 | BD Pharmigen | FITC | EV characterization | exosomes |
| CD81 | 555676 | BD Pharmigen | PE | EV characterization | exosomes |
| CD31 | 130-092-653 | Miltenyi | PE | EV characterization | Endothelial cells |
| ICAM | 559771 | BD Pharmigen | FITC | EV characterization | Endothelial cells and immune cells |
| VCAM | 551148 | BD Pharmigen | PE | EV characterization | Endothelial cells and immune cells |
| VE-cadherin | 560874 | BD Pharmigen | FITC | EV characterization | Endothelial cells |
| CD47 | 556044 | BD Pharmigen | FITC | EV characterization | Could act as a *don't eat me* signal to macrophages |
| TLR4 | ab11227 | Abcam | PE | EV characterization | inflammation |
| HLA class I | 560964 | BD Pharmigen | PE | EV characterization | Immune regulation |
| HLA class II | 347400 | BD Pharmigen | FITC | EV characterization | Immune regulation |
| HLA-DR | 555811 | BD Pharmigen | FITC | EV characterization | Immune regulation |
| CD44 | 555478 | BD Pharmigen | FITC | EV characterization | lymphocyte activation |
| CD279 (PD1) | 130-120-388 | Miltenyi | PE | EV characterization | Immune regulation |
| CD274 (PDL1) | 557924 | BD Pharmigen | PE | EV characterization, PBMC analysis | Immune regulation |
| CD11b | 562793 | BD Pharmigen | FITC | PBMC analysis | Monocyte activation |
| CD206 | 551135 | BD Pharmigen | FITC | PBMC analysis | M2 (immunosuppressive macrophages) differentiation |
| CD163 | 556018 | BD Pharmigen | PE | PBMC analysis | M2 (immunosuppressive macrophages) differentiation |
| CD4 | 317428 | BioLegend | PerCP/Cy5.5 | PBMC analysis | T reg marker |
| CD25 | 302606 | BioLegend | PE | PBMC analysis | Marker of proliferation, T reg marker |
| FoxP3 | 11-4777-42 | eBioscience | FITC | PBMC analysis | T reg marker |
| CD16 | 130-091-244 | Miltenyi | FITC | PBMC analysis | Natural killer cells, neutrophils marker |
| CD8 | 130-080-601 | Miltenyi | FITC | PBMC analysis | Marker of cytotoxic T cells |
| CD45 | 345809 | BD Pharmigen | PerCP | EV characterization | Lymphocytes |
