# Supplementary figures and images for: Extracellular Vesicles Released by Tumor Endothelial Cells Spread Immunosuppressive and Transforming Signals Through Various Recipient Cells
Source: Front Cell Dev Biol. 2020 Sep 9;8:698. doi: 10.3389/fcell.2020.00698 (PMC7509153; doi:10.3389/fcell.2020.00698)

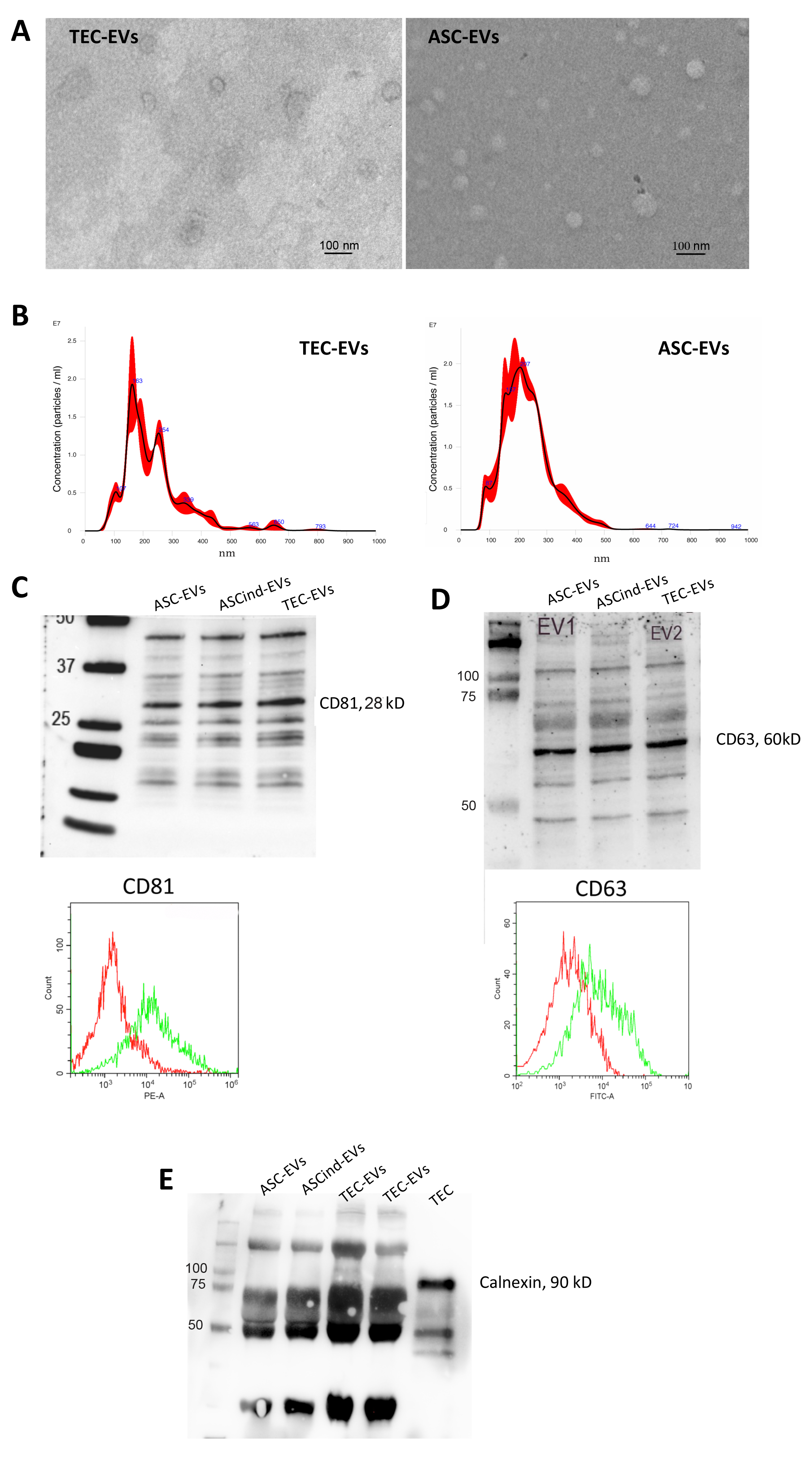

Supplement: Supplementary file 2 [file Image_1.TIF]

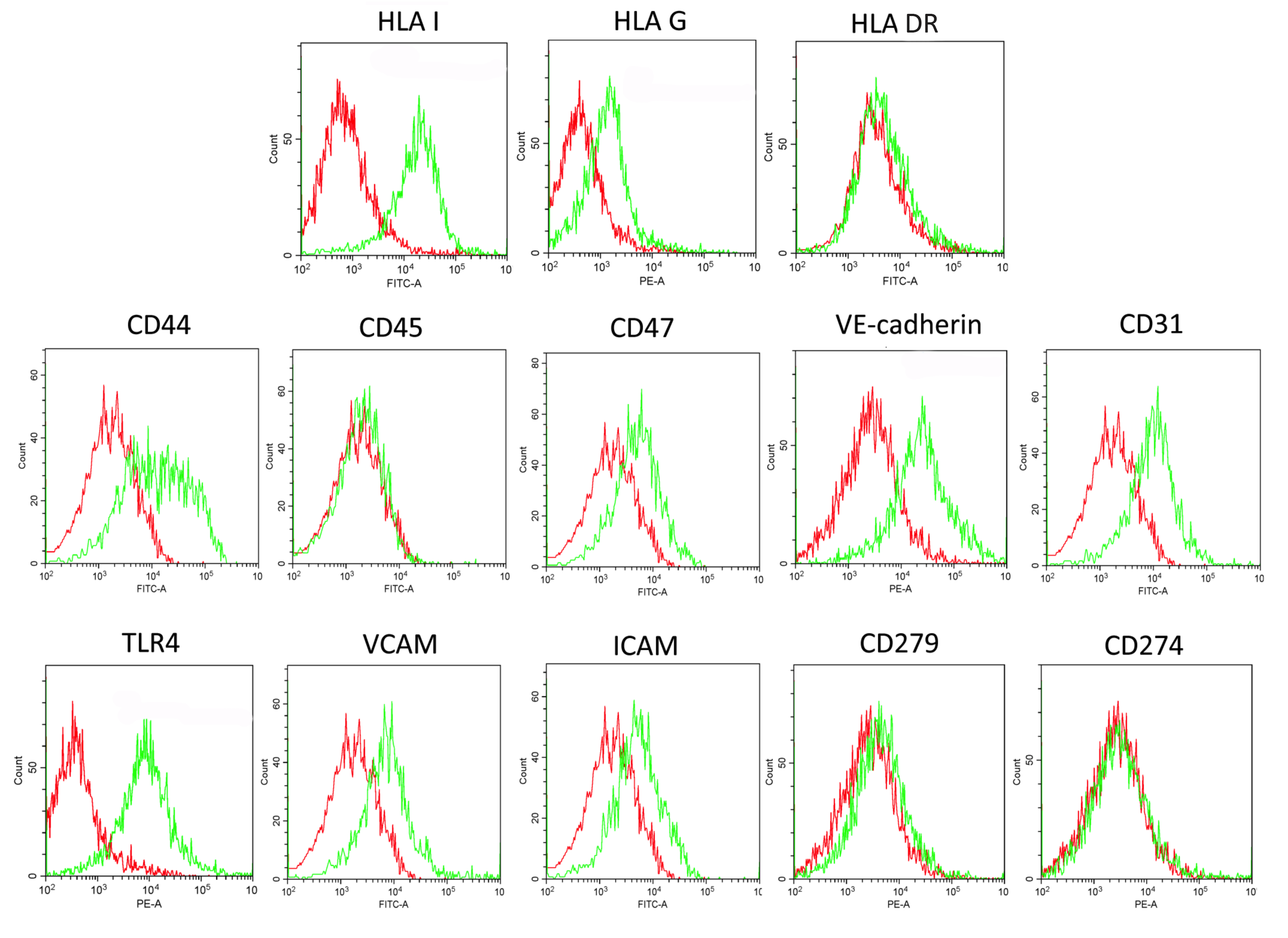

Supplement: Supplementary file 3 [file Image_2.TIF]

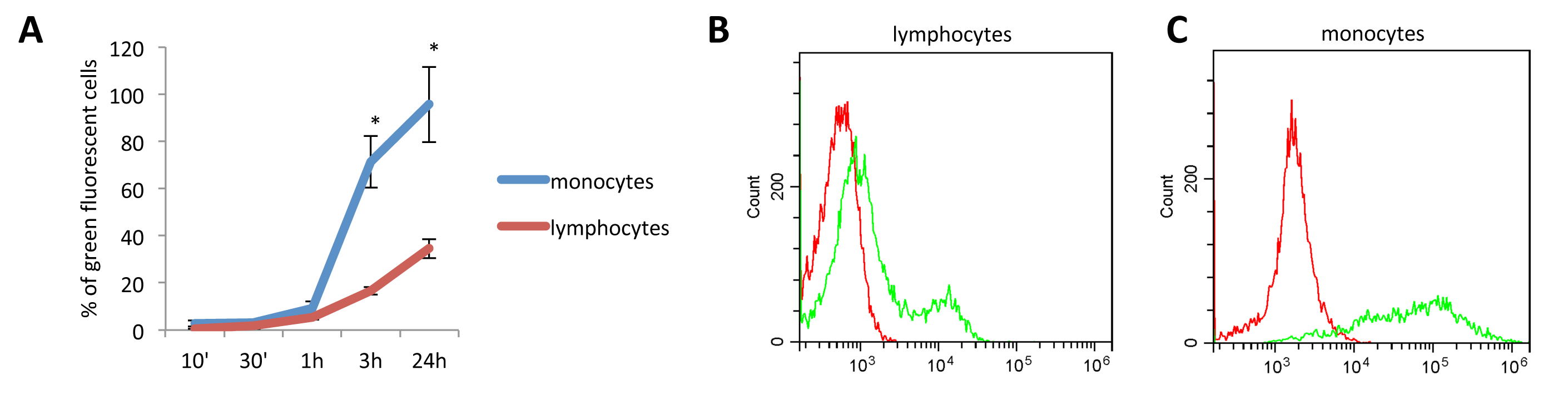

Supplement: Supplementary file 4 [file Image_3.TIF]

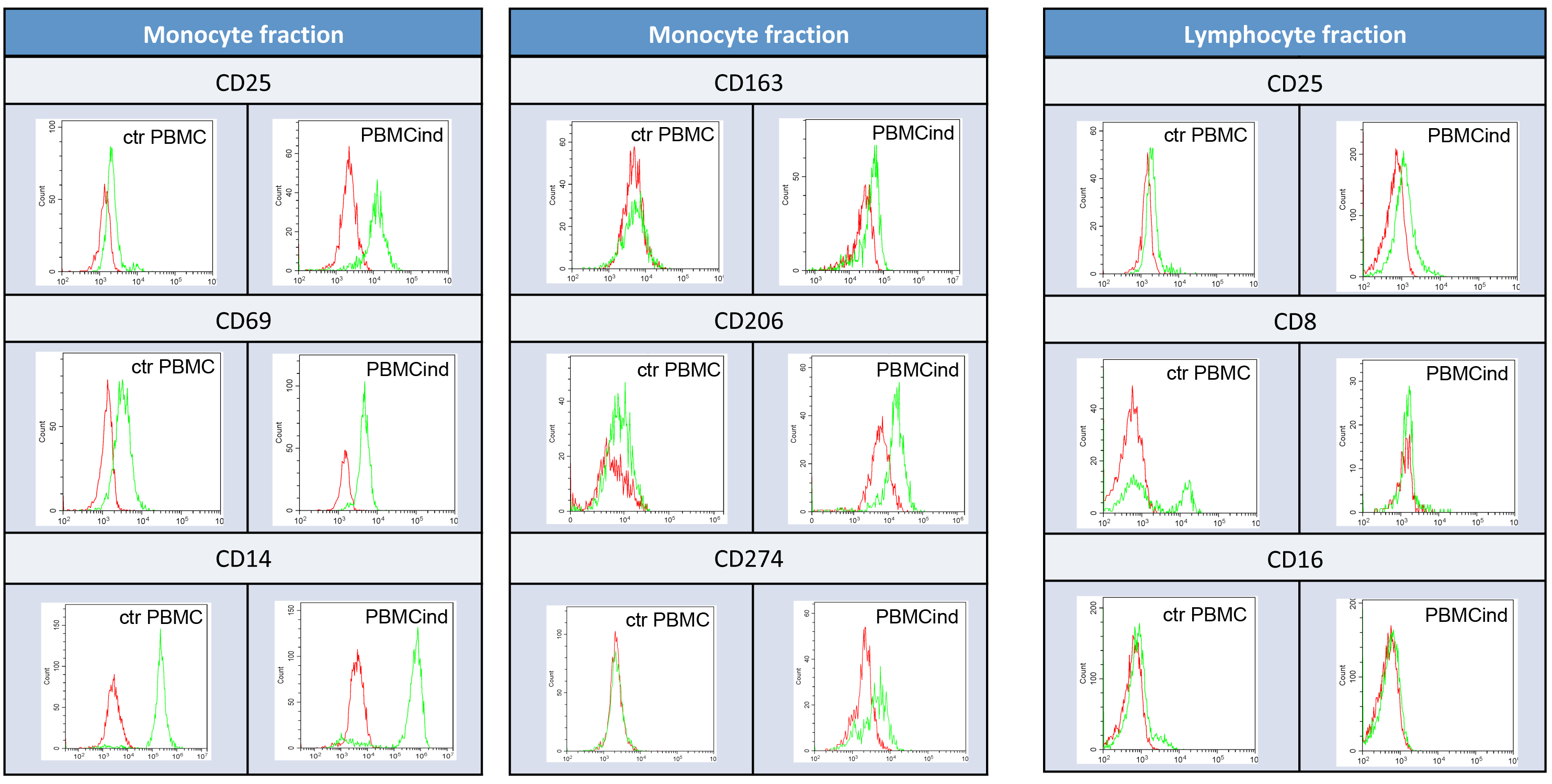

Supplement: Supplementary file 5 [file Image_4.TIF]

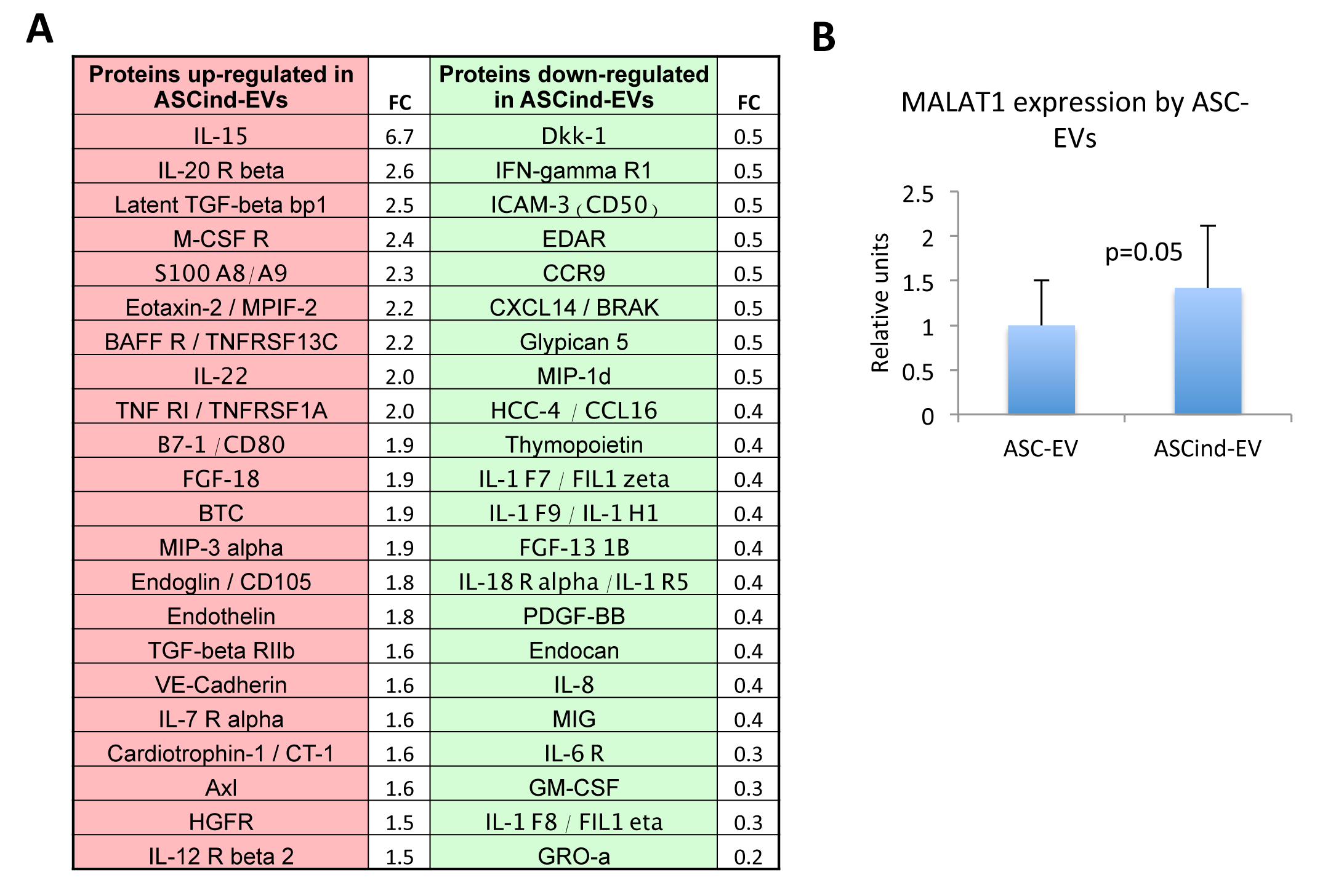

Supplement: Supplementary file 6 [file Image_5.TIF]

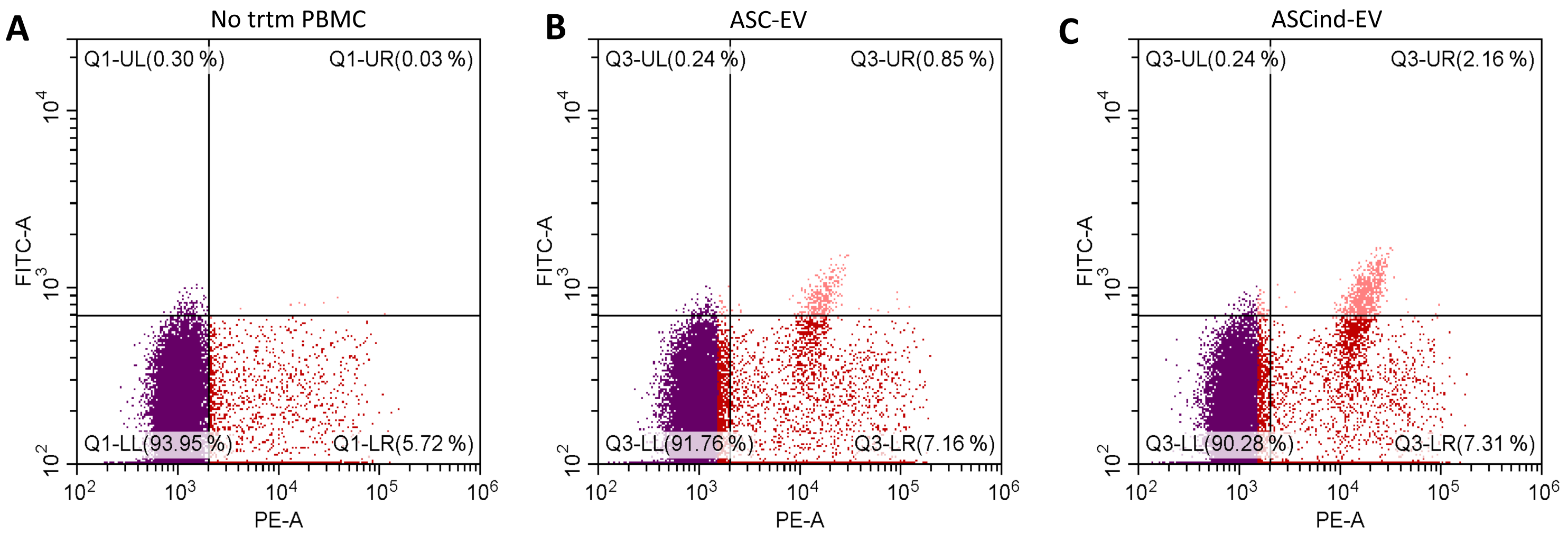

Supplement: Supplementary file 7 [file Image_6.TIF]
